# Pain Relief during Oocyte Retrieval by Transcutaneous Electrical Acupoint Stimulation: A Single-Blinded, Randomized, Controlled Multicenter Trial

**DOI:** 10.1155/2020/3285648

**Published:** 2020-09-22

**Authors:** Li Tian, Xiaojun Feng, Rong Zhang, Shuyu Wang, Rong Li, Rui Kong, Yuan Fan, Xiaoyu Zhang, Liying Zhou, Shuo Yang, Yin Yao, Yifan Bu, Yonglian Lan, Songping Han, Jisheng Han, Wei Sun

**Affiliations:** ^1^Center of Reproductive Medicine, Peking University People's Hospital, Beijing, China; ^2^Center of Reproductive Medicine, The Second Hospital Affiliated to Shandong University of Traditional Chinese Medicine, Jinan, China; ^3^Neuroscience Research Institute, Peking University, Department of Neurobiology, School of Basic Medical Sciences, Peking University Health Science Center, Key Lab for Neuroscience, The Ministry of Education, The Ministry of Health, Beijing, China; ^4^Department of Integration of Chinese and Western Medicine, School of Basic Medical Sciences, Peking University, Beijing 100191, China; ^5^Center of Reproductive Medicine, Beijing Obstetrics and Gynecology Hospital, Beijing, China; ^6^Center of Reproductive Medicine, Department of Obstetrics and Gynecology, Peking University Third Hospital, Key Laboratory of Assisted Reproduction, Beijing Key Laboratory of Reproductive Endocrinology and Assisted Reproductive Technology, Ministry of Education, Beijing, China; ^7^Wuxi Shen Ping Xin Tai Medical Technology Co., Ltd., Wuxi, China

## Abstract

Acupuncture has pain-relief effects, but no data were available on the use of transcutaneous electric acupoint stimulation (TEAS) in pain relief during oocyte retrieval. This study was designed to examine the effect of TEAS for pain relief in women undergoing transvaginal ultrasound-guided oocyte aspiration. This single-blinded, multicenter, randomized controlled trial was performed in China between May 2013 and May 2015. The subjects were randomized to mock TEAS and TEAS. TEAS or mock TEAS was administered 30 min before oocyte retrieval until the end of the operation. The primary and secondary endpoints were the pain measured using the visual analog scale (VAS) within 1 min and 1 hour after oocyte retrieval, respectively. Serum *β*-endorphin levels were tested in the first 50 patients/group. 390 women were undergoing oocyte retrieval. Pain levels evaluated using VAS within 1 min (18.6 ± 1.3 vs. 24.4 ± 1.7, *P* < 0.01) and 1 h after oocyte aspiration (4.6 ± 0.7 vs. 6.8 ± 0.8, *P* < 0.05) were lower in the TEAS group than in the mock TEAS group. Nausea assessment revealed a significantly lower VAS score in the TEAS group within 1 min (1.2 ± 0.4 vs. 2.9 ± 0.7, *P* < 0.033). Serum *β*-endorphin levels were significantly higher in the TEAS group than in the mock TEAS group (11.4 ± 0.5 vs. 9.1 ± 0.4, *P* < 0.001) after retrieval. Serum *β*-endorphin levels were higher in the TEAS group after the procedure than baseline (11.4 ± 0.5 vs. 9.1 ± 0.3, *P* < 0.001). Oocyte retrieval causes pain and discomfort, but TEAS is effective and safe for suppressing the pain and alleviating nausea associated with the operation.

## 1. Introduction

Transvaginal ultrasound-guided oocyte aspiration is a standard procedure in the process of in vitro fertilization and embryo transfer (IVF-ET). Oocyte aspiration is associated with pain and negative emotions. The level of pain perceived during oocyte aspiration varies widely from one individual to another and is often associated with the level of menorrhagia [1, 2].

Conscious sedation is the most commonly used method to reduce pain during oocyte aspiration (or ovum pick-up (OPU)) [3]. Conventional medical analgesia (CMA) during OPU includes sedative premedication with benzodiazepine, local analgesics administered as a paracervical block (PCB), and intravenous administration of fast-acting opiates during OPU. The ideal analgesia during OPU should guarantee adequate pain relief as well as rapid onset and recovery with minimum effects on the oocytes. Alfentanil and lidocaine have been detected in the human follicular fluid during OPU shortly after injection [4, 5]. The potential harm of these agents in follicular fluid on the development of embryos is unknown.

Kwan et al. showed that the simultaneous use of more than one method seems to achieve better pain relief than any modality alone [6]. Acupuncture, a treatment dating back at least 2500 years in China, has pain-relief effects [6–9]. In order to increase the reproducibility of this acupuncture-like technique and to reduce invasiveness, we chose to use transcutaneous electric acupoint stimulation (TEAS), a user-friendly technique, instead of using manual needling or electroacupuncture. In TEAS, the skin electrodes are placed on top of acupoints instead of inserting needles into the acupoints as in acupuncture or electroacupuncture therapy [10]. A similar analgesic effect has been shown to be produced by TEAS and electroacupuncture [11]. TEAS has been shown to possess analgesic effects or to potentiate standard analgesics, reducing the required doses [12–16]. TEAS technique provides a stable constant current output between 10 and 20 mA (at or higher than two times threshold intensity) at frequencies alternating between 2 and 100 Hz with 3 seconds each. This combination of output parameters is believed to be able to deliver the maximum analgesic effect [10].

We hypothesized that TEAS could be used to alleviate pain during oocyte retrieval. The aim of this single-blinded, multicenter, randomized controlled clinical trial was to examine the pain-relief effect of TEAS during OPU. The results could provide new analgesia methods for OPU, with a smaller use of drugs.

## 2. Materials and Methods

### 2.1. Study Design and Patients

This clinical trial was approved by the Institutional Review Board of Peking University (IRB00001052-13003) and the four participating centers, and it conforms to the provisions of the Declaration of Helsinki (as revised in Tokyo 2004). Written informed consent was obtained from all subjects. The trial was registered prior to patient enrollment at the Chinese Clinical Trial Registry (ChiCTR-TRC-13003952, Principal investigator: Jisheng Han, Date of registration: 2013-11-25).

This was a single-blinded, multicenter, randomized controlled clinical trial conducted in ethnic Chinese who sought assisted reproductive technology (ART) at the reproductive centers of Peking University People's Hospital, Peking University Third Hospital, Beijing Obstetrics and Gynecology Hospital, and Shandong University of Traditional Chinese Medicine Second Affiliated Hospital. The study was completed between May 2013 and May 2015. Information regarding the study was provided to all patients during their initial IVF consultation. This manuscript adheres to the applicable CONSORT guidelines.

The inclusion criteria were (1) infertile women of 20–45 years of age; (2) those undergoing microstimulated superovulation, natural cycle; and (3) those with five or fewer oocytes (diameter >10 mm) retrieved. The exclusion criteria were (1) use of sedative or analgesic drugs or (2) use of a pacemaker, any device to assist the heart or lungs, Holter monitoring, or any implanted device sensitive to microwaves.

### 2.2. Randomization and Mask

Participants were randomly assigned (in a 1 : 1 ratio) to receive either TEAS or mock TEAS. Randomization was stratified according to the different centers. A third-party statistician produced a randomization table with stratified blocks of four using SAS 9.13 (SAS Institute, Cary, NY, USA) and prepared sequential sealed opaque envelopes. The women undergoing OPU and the physician who evaluated the levels of pain, nausea, and vomiting were blinded to grouping. The evaluation was performed by one physician at each center who received unified training. The operators who did TEAS treatment were not blinded since the intensity of the stimulation in the treatment group needs adjustment, and small muscle trembling near the stimulation sites were visible.

### 2.3. Oocyte Retrieval

All patients received a standard microstimulated superovulation protocol. On the third day of the menstrual cycle, 300 units HMG225 was used to promote the ovum and was continuously used for 8 to 10 days. Follicle growth was evaluated by ultrasound before hCG administration. Intramuscular injection of human chorionic gonadotropin (hCG; 10,000 IU) was performed when the maximum follicle reached at least 18 mm in diameter. Oocyte retrieval was scheduled 36 h later.

### 2.4. Intervention

For women allocated to the TEAS treatment group, TEAS (HANS-200A; Nanjing Jisheng Medical Technology Company, Nanjing, China) was administered 30 min before OPU and continued during the OPU procedure till the operation was completed.

LI 4 (Hegu at the part between the thumb and index figure) and PC 8 (Laogong at the palm) on one side, PC 6 (Neiguan), and SJ 5 (Waiguan) on the wrist of the other side received sticking skin electrodes of 4 × 4 cm. The TEAS was administered at alternating frequencies (2 Hz for 3 s and 100 Hz for 3 s, 2/100 Hz), with a pulse width of 0.6 ms at 2 Hz and 0.2 ms at 100 Hz and an intensity of 10–20 mA (two times the threshold intensity) [8]. The TEAS treatment started 30 min before OPU and ended after the procedure was completed. The duration of a typical operation was <15 min.

For women in the mock TEAS group, 2/100 Hz intermittent (10 s on and 20 s off) TEAS at 5 mA was given (slightly above the threshold). The acupoints used were the same as for the TEAS group. The mock TEAS represents a weak (minimal), but still, sensible stimulation, which has been demonstrated as a successful placebo (psychologically effective yet physiologically near inert) treatment modality in a previous smoke abatement trial [17].

Body temperature, respiratory rate, heart rate, and blood pressure were measured before OPU. If the patients changed their minds before the operation and demanded the use of analgesics, those patients were naturally excluded. Intraoperatively, if patients reported unbearable pain, propofol 2 mg/kg was immediately administered. The specific conditions of this event for those patients were recorded; they were not included in the final statistical population. Patients with postoperative pain received sedatives or analgesics (such as morphine pump) immediately after the first postoperative evaluation; they were included in the statistical analysis.

### 2.5. Endpoints

The primary endpoint was the visual analog scale (VAS) for pain within 1 min after OPU. The secondary endpoint was the VAS for pain at 1 h after OPU. The occurrence of nausea and vomiting within 1 min and 1 h after OPU was evaluated as safety endpoint using a VAS, where 0 indicates no pain or nausea and 100 indicates the worst pain or nausea ever imagined. Vomiting was also recorded. The reproductive outcomes were exploratory endpoints.

### 2.6. Blood Sampling and Serum *β*-Endorphin Measurements

Blood tests were performed for the first 50 patients in each group at Shandong Center. Samples were collected by trained nurses before and within 1 min after OPU. Patients were fasted overnight and allowed for only moderate amounts of drinking water. Venous blood (2 mL) was collected into clotting tubes. The samples were placed at room temperature for 30 min and then centrifuged at 1600 × g for 15 min at 4°C. The serum was isolated, divided into 600 *μ*L aliquots, and immediately frozen at −80°C. Serum concentrations of *β*-endorphin were measured using a commercially available human *β*-endorphin ELISA kit (#E01E0181, E01E0181).

### 2.7. Adverse Events

Adverse events (AEs) were followed by the investigators until 1 week later after the intervention.

### 2.8. Statistical Analysis

The sample size was estimated based on data collected from a pilot study in which the VAS score was 3.0 ± 1.2 for the TEAS group and 4.9 ± 1.5 for the control group. *α* was set 0.05, and the power was set 0.8. According to the formula NC=NT=(2(*Z*_1−*α*_+*Z*_1−*β*_)^2^*σ*^2^)/(Δ_−*δ*_)^2^, at least 157 patients were needed in each group according to 1 : 1. The estimated dropout rate was 20%, and then the sample size of each group was 196 patients or 392 patients total.

Data were analyzed using SPSS 19.0 (IBM, Armonk, NY, USA). Continuous data were tested for normal distribution using the Kolmogorov–Smirnov test. Normally distributed continuous variables are presented as means ± standard deviation and were analyzed using the Student *t*-test. The paired *t*-test was used for intragroup analyses. Continuous data with a skewed distribution were presented as medians (range) and were analyzed using the Mann-Whitney *U* test. Categorical data were presented as frequencies and were analyzed using the chi-square test. The primary endpoint compared between the two groups was using the Student *t*-test. Two-sided *P* values <0.05 were considered statistically significant.

## 3. Results

### 3.1. Patients

**Figure 1:**
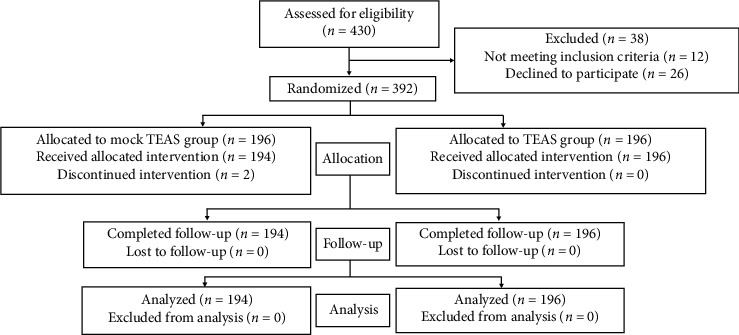
CONSORT 2010 flow diagram of the patients who underwent oocyte retrieval.

A total of 430 subjects were recruited between May 2013 and May 2015 (Figure 1). Subjects with more than five oocytes retrieved were excluded (*n* = 12). A total of 392 subjects were randomized, and 390 subjects were analyzed, including 194 in the mock TEAS group and 196 in the TEAS group. All patients were followed up. The intent-to-treat (ITT) principle was used to analyze the data. As shown in Table 1, there were no significant intergroup differences in age, body mass index (BMI), duration of infertility, the reason for infertility, vital signs, numbers of oocytes, or other variables.

### 3.2. Primary Endpoint

**Figure 2:**
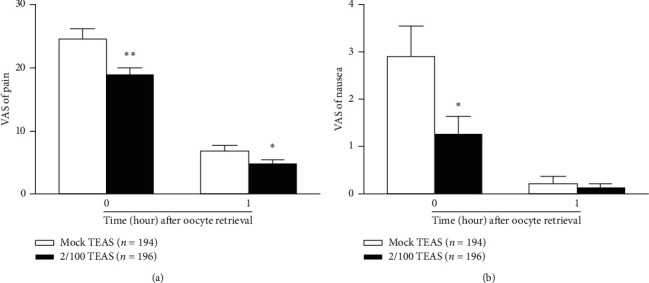
Effects of transcutaneous electrical acupoint stimulation (TEAS) on visual analog scale (VAS) scores of pain, nausea, and vomiting within 1 min and 1 h after oocyte retrieval. TEAS significantly decreased the VAS scores of pain (a) and nausea (b) in the TEAS group. Data are shown as mean ± standard deviation. ^*∗∗*^*P* < 0.01, ^*∗*^*P* < 0.05 compared with the mock TEAS group (unpaired two-tailed Student's *t*-test).

As shown in Figure 2(a), pain levels were less severe in the TEAS group than in the mock TEAS group both within 1 min (18.6 ± 1.3 vs. 24.4 ± 1.7, *P* < 0.01) and 1 h (4.6 ± 0.7 vs. 6.8 ± 0.8, *P* < 0.05) after OPU.

### 3.3. Safety Endpoints

Nausea assessed by the VAS within 1 min after oocyte aspiration revealed a significantly lower nausea level in the TEAS group (1.2 ± 0.4) than in the mock TEAS group (2.9 ± 0.7, *P*=0.033). There were no significant differences between the two groups in nausea 1 h after OPU (0.1 ± 0.1 vs. 0.2 ± 0.2, *P*=0.59) (Figure 2(b)), which is likely due to the diminishing symptoms and the very low level of vomiting 1 h after the procedure.

Six patients in the mock TEAS group and two patients in the TEAS group vomited within 1 min after OPU, without differences in VAS scores between the two groups (data not shown). No patient vomited 1 h after the procedure.

After 1 week of follow-up, no severe AEs were found.

### 3.4. Serum *β*-Endorphin Levels

**Figure 3:**
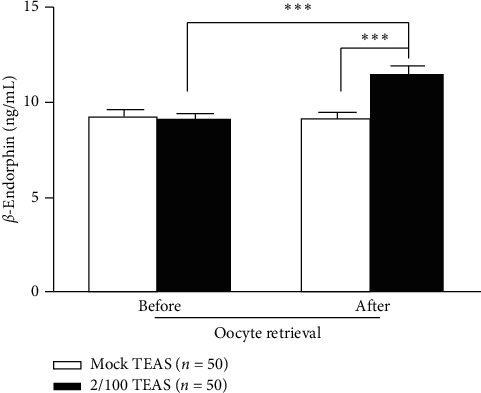
Changes in serum *β*-endorphin levels in the women who underwent oocyte retrieval after transcutaneous electrical acupoint stimulation (TEAS) intervention. The serum levels of *β*-endorphin after oocyte retrieval were significantly higher in the TEAS group compared with the mock TEAS group (unpaired two-tailed Student *t*-test). The serum levels of *β*-endorphin after oocyte retrieval were higher than that at baseline in the TEAS group (paired two-tailed Student's *t*-test). Data are expressed as means ± standard deviation. ^*∗∗∗*^*P* < 0.001.

As shown in Figure 3, serum *β*-endorphin levels were significantly higher in the TEAS group compared with the mock TEAS group (11.4 ± 0.5 vs. 9.1 ± 0.4, *P* < 0.001) after OPU. Serum *β*-endorphin levels were higher in the TEAS group after the procedure than before (11.4 ± 0.5 vs. 9.1 ± 0.3, *P* < 0.001). No change was found before and after oocyte retrieval in the mock TEAS group.

There were no significant differences between the mock TEAS and TEAS groups regarding the reproductive outcomes (Supplementary Table S1).

## 4. Discussion

In the present study, pain (within 1 min and 1 h after) and nausea (within 1 min after OPU) were significantly alleviated by TEAS compared with the mock TEAS intervention. The novelties of the present study were to use TEAS instead of EA and to try TEAS in the setting of a multicenter study. Concerning the design of the control group, since TEAS stimulation would produce subjective feeling, it is very difficult to design a satisfactory placebo group. Based on a previous study in smoking cessation [17], we used a mock TEAS design with minimal but still sensible TEAS stimulation. In order to reduce further the therapeutic effect of the mock stimulation, the stimulator was set to an intermittent mode with 10 s on and 20 s off, thus shortening the duration of stimulation to one-third of the TEAS treatment group. This study suggests promising prospects for the use of TEAS during OPU.

Indeed, previous studies showed that EA or TEAS reduces patient-controlled analgesia (PCA) requirement and incidence of nausea, vomiting, dizziness, and pruritus [18, 19] and accelerates surgical recovery [15]. Stener-Victorin et al. [20, 21] were the first to study the analgesic effect of EA on oocyte retrieval; compared with the alfentanil group, the patients in the acupuncture group had less abdominal pain and other pains. There was also less nausea and stress 2 h after the operation [21]. Similar results were obtained by Zhang et al. [22] using EA stimulation at bilateral LI 4 (Hegu).

The output parameters used in EA or TEAS therapy appear to be critical for achieving the optimal efficacy of TEAS. Enkephalins are preferentially released in the brain in response to a 2 Hz stimulation, and dynorphin release is promoted preferentially by a 100 Hz stimulation in the spinal cord. Therefore, the alternating 2 and 100 Hz stimulation with a dense disperse pattern has been demonstrated to be most effective in activating the endogenous opioid system [10]. Stener-Victorin et al. [20, 21] explored the frequency specificity of EA for pain relief in OPU and found no significant difference between the mixed frequency (2/80 Hz) and 20 Hz. This was different from the findings by Han [10] in which stimulation at alternating 2/100 Hz produced a stronger analgesic effect than either a single low frequency (2 Hz) or a high frequency (100 Hz). Looking into the details of Stener-Victorin et al.'s study [20] and Gejervall et al.'s study [23], it was found that 2 Hz of EA stimulation was used on the hand and 100 Hz [20] or 80 Hz [23] on the abdomen (referred to as “2 + 100” or “2 + 80” stimulation). For the high-frequency stimulation, it was continuous at 80 Hz with a pulse duration of 0.180 ms. Nevertheless, the “low-frequency (2 Hz)” stimulation was actually in the intermittent mode with 80 Hz stimulation delivered in bursts (burst frequency: 2 Hz, burst duration: 100 ms). In both groups, the frequency of stimulation was the same (80 Hz) but delivered in different modes.

A better analgesic effect of using the alternating 2/100 Hz stimulation for pain relief was supported by two recent studies [24, 25]. They also provided an additional novel mechanism other than the synergism between enkephalin release and dynorphin release proposed by Han [10]. TEAS at 2/100 Hz significantly reduced intraoperative opioid dosage as well as the pain score, extubation time, and postanesthetic ICU (PACU) stay, compared with the three other groups [25]. In the present study, TEAS was administered at alternating frequencies (2 Hz for 3 s and 100 Hz for 3 s, 2/100 Hz), with a pulse width of 0.6 ms at 2 Hz and 0.2 ms at 100 Hz and an intensity of 10–20 mA, and a significant analgesic effect was achieved. Nevertheless, a single TEAS protocol was used, and future studies should compare multiple approaches within the same study.

The selection of acupoints used in the present study was based on reports in the literature that LI 4 (Hegu) is an acupoint most commonly used for analgesia [22] and PC 6 (Neiguan) is commonly used in treating nausea and vomiting [26, 27]. For the selection of the duration of TEAS intervention, we referred to the findings obtained in manual needling that 30 min of stimulation seems to be the minimal requirement for the full expression of the analgesic effect [28]. It was also reported by Humaidan and Stener-Victorin [29] and Yao et al. [15] that the analgesic effect produced by a brief acupuncture intervention for a few minutes [29] was not as good as those applied for 30 min before the operation [15]. In a recent study, Liodden et al. [30] demonstrated that no beneficial effect for pain control was observed if acupuncture was delivered only after the start of general anesthesia in a surgical procedure and not before anesthesia. In the present study, no general anesthesia was administered.

Several underlying mechanisms have been proposed for the EA/TEAS-induced reduction of postoperative nausea and vomiting. In a clinical setting, TEAS apparently relieves nausea and vomiting during and after cesarean section [31], and the mechanism seems to be associated with decreased plasma 5-HT concentration [32]. Aside from serotonin, neuropeptide Y (NPY) was also suggested to be involved in the nausea-reducing effect of EA/TEAS. Stener-Victorin et al. [21] reported that EA induces an increase in NPY concentration in the follicular fluid of the ovary in women undergoing OPU, while cancer-related nausea is known to be accompanied by a reduced NPY function [33].

*β*-Endorphin is a hormone mainly used by the human body to control stress and pain and is involved in reward recognition [34]. In the present study, the serum *β*-endorphin levels were significantly increased by TEAS and were higher in the TEAS group compared with the mock TEAS group after OPU. *β*-Endorphin probably played a role in the reduction of pain by TEAS. This effect of TEAS on *β*-endorphin levels was observed by the early studies of TEAS [35–37].

TEAS is generally considered to be a safe procedure [12, 38, 39]. No adverse events were reported in the present study.

The present study has limitations. Considering the characteristics of the TEAS procedure, the TEAS operator cannot be blinded. Only the patients and evaluators were blinded. Only *β*-endorphins were measured, but additional biomarkers of pain and stress could be measured in future studies. The conventional medical analgesia (CMA) group would have been useful for fully evaluating the efficacy of TEAS. Unfortunately, a CMA group was not included in the original design of this randomized controlled trial. Since the trial is completed, it cannot be provided. Such a group will be included in future trials.

## 5. Conclusions

In conclusion, the present study demonstrates that the pain associated with OPU was less in the TEAS group compared with the mock TEAS group. The nausea was also less severe in the TEAS group than in the mock TEAS group. These findings provide strong evidence for the clinical efficacy of TEAS in reducing pain and discomfort associated with OPU.

## Figures and Tables

**Table 1:** Characteristics of the patients.

Characteristics	Mock TEAS (*n* = 194)	TEAS (*n* = 196)	*P*
Age (years)	37.0 ± 5.5	36.7 ± 5.6	0.594
BMI (kg/m^2^)	22.9 ± 3.0	23.2 ± 3.4	0.356
Duration of infertility (years)	5.6 ± 4.5	5.1 ± 3.5	0.221
Reason for infertility, *n* (%)	—	—	0.575
Male	23 (11.9)	19 (9.7)	—
Female	141 (72.7)	140 (71.4)	—
Both	30 (15.5)	37 (18.9)	—
Body temperature (^o^C)	36.4 ± 0.2	36.4 ± 0.2	>0.999
Respiratory rate (per min)	27.1 ± 21.0	28.9 ± 23.1	0.421
Heart rate (per min)	67.2 ± 20.6	65.8 ± 22.3	0.520
Systolic pressure (mmHg)	111 ± 8	111 ± 8	>0.999
Diastolic pressure (mmHg)	72 ± 6	73 ± 6	0.101
Number of oocytes retrieved	2.6 ± 1.5	2.8 ± 1.6	0.204

BMI: body mass index.

## Data Availability

The data used to support the findings of this study are available from the corresponding author upon request.

## Supplementary Materials

Supplementary Materials Table S1: comparison of reproductive outcomes between the mock TEAS and TEAS groups.
