# Supplementary material for: Pain Relief during Oocyte Retrieval by Transcutaneous Electrical Acupoint Stimulation: A Single-Blinded, Randomized, Controlled Multicenter Trial
Source: Evid Based Complement Alternat Med. 2020 Sep 22;2020:3285648. doi: 10.1155/2020/3285648 (PMC7530499; doi:10.1155/2020/3285648)
Supplement: Supplementary Materials — Table S1: comparison of reproductive outcomes between the mock TEAS and TEAS groups. [file 3285648.f1.docx]

**Supplementary Table S1**. Comparison of reproductive outcomes between the mock TEAS and TEAS groups.

|  | Mock TEAS (n=196) | TEAS (n=194) | P |
| --- | --- | --- | --- |
| Number of oocytes, mean ± SD | 2.6±1.5 | 2.8±1.6 | 0.165 |
| Number of patients underwent ET, % | 65.3 (128/196) | 62.9 (122/194) | 0.618 |
| Biochemical pregnancy rate, % | 42.2 (54/128) | 32.8 (40/122) | 0.125 |
| Clinical pregnancy rate, % | 37.3 (50/128) | 30.3 (37/122) | 0.147 |
| Early pregnancy loss (among those with a biochemical pregnancy), % | 25.9 (14/54) | 17.5 (7/40) | 0.332 |
| Baby took home rate, % | 25.8 (33/128) | 21.3 (26/122) | 0.405 |

In the mock TEAS group, 65.3% (128/196) of the patients underwent embryo transplantation. Among them, 42.2% (54/128) of the patients achieved a biochemical pregnancy, and 37.3% (50/128) of the patients achieved a clinical pregnancy. In the patients with a biochemical pregnancy, 25.9% (14/54) of the patients had an early pregnancy loss. In the patients who underwent embryo transplantation, 25.8% (33/128) took their baby home.

In the TEAS group, 62.9% (122/194) of the patients underwent embryo transplantation. Among them, 32.8% (40/122) of the patients achieved a biochemical pregnancy, and 30.3% (37/122) of the patients achieved a clinical pregnancy. In the patients with a biochemical pregnancy, 17.5% (7/40) of the patients had an early pregnancy loss. In the patients who underwent embryo transplantation, 21.3% (26/122) took their baby home.

The “number of oocytes” variable was found to follow the normal distribution based on the Kolmogorov-Smirnov test; it was presented as means ± standard deviation and was analyzed using the Student t-test. Categorical data are presented as frequencies and were analyzed using the chi-square test.

Abbreviations: TEAS: transcutaneous electric acupoint stimulation; SD: standard deviation; ET: embryo transplantation.
